# Participation and autonomy, independence in activities of daily living and upper extremity functioning in individuals with spinal cord injury

**DOI:** 10.1038/s41598-024-59862-2

**Published:** 2024-04-20

**Authors:** Lamprini Lili, Katharina S. Sunnerhagen, Tiina Rekand, Margit Alt Murphy

**Affiliations:** 1 Department of Clinical Neuroscience, Institute of Neuroscience and Physiology, Sahlgrenska Academy, University of Gothenburg, Vita Straket 12, plan 4, 41346 Gothenburg, Sweden; 2 Department of Neurological Rehabilitation, Sahlgrenska University Hospital, Gothenburg, Sweden; 3 Department of Neurology, Haukeland University Hospital, Bergen, Norway; 4 Department of Occupational Therapy and Physiotherapy, Sahlgrenska University Hospital, Gothenburg, Sweden

**Keywords:** Spinal cord injury, Autonomy, Participation, Independence, Activities of daily living, Upper extremity, Kinematics, Outcomes research, Spinal cord diseases

## Abstract

Improvements in care and rehabilitation have resulted in a higher proportion of people living with spinal cord injury (SCI), which calls for an increased focus on participation and autonomy. This observational cross-sectional study investigated the impact of SCI on autonomy and how it correlates to activity performance and upper extremity functioning. A total of 25 adults (mean age 58 years) with chronic cervical or thoracic SCI were included. Self-perceived autonomy was measured with Impact on Participation and Autonomy questionnaire, independence in activities of daily living (ADL) with Spinal Cord Independence Measure, upper extremity functioning with Action Research Arm Test (ARAT) and kinematic measures of the drinking task. The results showed that most participants perceived injury-related restrictions in outdoor autonomy (80%), family role (76%), and in indoor autonomy (72%). Independence in self-care (r = 0.72), mobility (r = 0.59) and upper extremity kinematics of movement time (r = 0.63) and smoothness (r = 0.49) were correlated to indoors autonomy. Social life autonomy was correlated to self-care (r = 0.50) and ARAT (r = 0.41). In conclusion, autonomy was perceived restricted after SCI in several major life areas and correlated with independence in ADL and upper extremity functioning. The aspects of autonomy should be considered more in goal setting and clinical decision-making.

## Introduction

Participation, defined as involvement in life situations, including autonomy and societal roles, according to the International Classification of Functioning, Disability and Health (ICF)^1,2^, is a fundamental right of individuals with disabilities^3^ and correlates positively with well-being, patient satisfaction, and quality of life^4^. Consistent with individual expectations and values, participation in different life areas is considered as the ultimate goal of rehabilitation^1,5,6^. Participation incorporates a social perspective on life activities and is perceived by a person or observed by others^7^. Participation can be achieved either actively, through activities that the person performs him/herself (executional), or passively, by delegating others to perform the activities according to his or her wishes and instructions (decisional)^8^. A fundamental pre-requisite for participation is the capacity for self-governance, also called autonomy^5,6^. The highest level of participation can be achieved by regaining and maintaining the highest possible level of autonomy^5^. Independence is another term commonly used along with autonomy, but there is a distinct difference between these terms. Independence indicates a person’s ability to perform daily activities with little or no help from others, while the autonomy refers to a person’s ability to choose how, when and by what means the activities will be performed according to their preferences^5,9^.

Recent improvements in care and rehabilitation, resulting in a higher proportion of people living longer with the consequences of spinal cord injury (SCI) in the society, redirect the focus of care from bodily impairments towards participation in life roles and activities^6^. The role of upper extremity is essential in most of the activities of daily living (ADL), as also reflected in rehabilitation priorities selected by the individuals with SCI^10^. SCI induced upper extremity impairment (body functions domain in ICF) correlate with limitations in activity capacity as well as activity performance in a real-life environment (activity domain in ICF)^11–15^. However, less is known about potential relationships between upper extremity functioning and the self-perceived autonomy (participation domain in ICF) after SCI. A deeper understanding of the potential relationships across different ICF domains in the context of the upper extremity could support clinical decision-making, facilitate goal setting, and improve SCI rehabilitation^16^. Thus, this study aimed to investigate to what degree the SCI impacts the self-perceived autonomy and participation in major life areas and how the restrictions in autonomy correlate to the upper extremity functioning and activity performance in activities of daily living in people with cervical and thoracic SCI. In reference to ICF, we expected that activity performance and independence in ADL would show stronger relationships with autonomy than the upper extremity functioning.

## Methods

### Study design and participants

In this observational cross-sectional study, participants were recruited from an outpatient rehabilitation unit at Sahlgrenska University Hospital, Gothenburg, Sweden, in 2018. All available patients were screened for potential inclusion. Inclusion criteria were: cervical or thoracic neurological level of injury with all grades of severity except from AIS E according to the ASIA (American Spinal Injury Association) Impairment Scale (AIS)^17,18^, injury present for more than 1 year, age ≥ 18 years, not fully independent in ADL according to Spinal Cord Independence Measure (SCIM) total score (scoring less than maximum of 100 points), and ability to use the upper extremity to some degree for everyday tasks such as drinking from a glass. Exclusion criteria were difficulties in communicating in Swedish and other psychological, neurological, or musculoskeletal comorbidities that could affect upper extremity use in ADL. The neurological level of the SCI was determined according to the International Standards for Neurological Classification of Spinal Cord Injury (ISNCSCI) developed by the American Spinal Injury Association (ASIA) and the severity of the injury according to the ASIA Impairment Scale (AIS; A-E)^17,18^.

Informed and written consent was obtained from all participants prior to their inclusion in the study. The study was approved by the Swedish Ethical Review Authority (registration number 408–17) and conducted in accordance with the Declarations of Helsinki. Before the recruitment of participants, this study was registered at researchweb.org (https://www.researchweb.org/is/vgr/project/260901). The reporting of this study adheres to the guidelines of Strengthening the Reporting of Observational studies in Epidemiology (STROBE) statement^19^.

### Self-perceived autonomy in participation

The Impact on Participation and Autonomy (IPA) is a patient reported questionnaire emphasising the personal perspective in the fulfilment of life roles rather than the normative perspective of another person (e.g., a clinician)^20–22^. The IPA has been tested on adults with various conditions, including SCI^23–26^. In this study, the self-perceived autonomy was assessed with the translated Swedish version of the English version of IPA^21,23,25–27^.

The IPA questionnaire contains nine sections covering multiple aspects of life: mobility, self-care, activities in and around the house, looking after money, leisure, social life and relationships, helping and supporting others, paid or voluntary work, education and training. In addition to these nine sections, IPA contains a concluding question (question 10) on the impact on life in general ("My chances of living life the way I want to are"). The questions are expressed as “my chances of doing the activity, either by myself or others as I want are…” and the response options range from very good to very poor (0 to 4). The responses are categorised into five life impact areas: autonomy indoors, family roles, autonomy outdoors, social life and relationships, work and education. The *indoor autonomy* includes questions on indoor mobility and self-care; the *family role* includes questions on activities in and around the house and economy; the *outdoor autonomy* includes outdoor mobility, leisure time autonomy and general impact on life; the *social life* and relationships includes questions on social life, relationships and helping and supporting others; and the *work/education* domain includes paid and voluntary work, education and training (Supplementary table).

The IPA questionnaire also includes two optional open-ended questions. First, the respondent is asked to list the top three injury-related problems concerning the nine sections of the IPA questionnaire. Finally, the respondent is asked to add other aspects important for autonomy and participation ("Are there any other aspects you want to mention that we have not asked you about?").

### Activity performance in ADL

Activity performance in ADL was assessed by the Spinal Cord Injury Independence Measure (SCIM-III) administered by interview (SCI specialist physician)^28,29^. The SCIM assesses independence in ADL on 19 items divided into three subscales: self-care, respiration/ sphincter management and mobility. A total score of 100 indicates full independence. The self-care domain has shown strongest correlation with upper extremity functioning in people with SCI^11,13–15^.

### Upper extremity functioning

Upper extremity activity capacity was assessed using the Action Research Arm Test (ARAT)^30^ by researcher/physician (specialist in SCI). The ARAT has been increasingly used in individuals with SCI^31,32^ and has shown a good correlation with upper extremity impairment^12^ and activity performance^11^. ARAT includes 19 unimanual tasks hierarchically organised into 4 subscales (grasp, grip, pinch, and gross movement). The total score of 57 indicates a better capacity.

Upper extremity function during drinking task was measured by a 5-camera 3D kinematic analysis system (MCU240 Hz, Qualisys AB, Gothenburg, Sweden)^33^. The spherical reflective markers were attached to the body (third metacarpophalangeal joint, styloid process of ulna, lateral epicondyle of humerus, central acromion, the upper part of sternum, between the eyebrows), and on the drinking glass^33,34^. The data was filtered with a 6 Hz second-order Butterworth filter and analyzed in Matlab software (The Mathworks Inc, Natick, Ca).

The standardized kinematic analysis of the drinking task included 5 movement phases: reaching, moving the glass to the mouth, drinking a sip of water, moving the glass back on the table and returning the hand to the initial position^34,35^. The height of the chair and table was adjusted to ensure a standardized sitting position (90-degree angle in knee and hip, upper arm vertical, forearm horizontal, wrist aligned with the edge of the table, palm resting on the table). A hard plastic glass filled with 100 ml of water, was placed 30 cm away from the edge of the table in the midline of the body. After familiarization, the task was repeated unimanually in natural speed at least 5 times with each arm^35^. Data from the more affected arm (according to the ARAT score) or non-dominant arm (in case no difference in ARAT) was used in the analysis.

The kinematic variables of movement time, smoothness, wrist angle and trunk displacement were selected as key measures for SCI based on our previous research^33–35^. The start and end of movement time was defined from the 2% threshold of the maximum velocity. The number of movement units (defined as ≥ 20 mm/s amplitude and ≥ 150 ms between peaks) during the 4 movement phases (except the drinking phase) defined movement smoothness^34^. The minimal number of movement units for the drinking task is four, one for each movement phase. The wrist angle was the maximum dorsal flexion reached during the reaching and transport (phase 1 and 2). Trunk displacement was determined by the maximum displacement of the sternum marker during the entire task in sagittal plane.

### Statistical analysis

Statistical analyses were performed using the IBM Statistical Package for Social Sciences™ (SPSS, version 24). Descriptive statistics are reported for demographics, clinical characteristics and measures. For the responses of IPA questionnaire, the median value of each impact area was extracted for analysis. The top three SCI-related problematic life situations ranked by participants were independently linked by two authors (LL, MAM) to one of the most appropriate IPA "life impact area". A consensus was reached through a discussion with the third author (KSS). The frequency of the ranked impact areas and problematic aspects were calculated.

Due to the non-normal distribution of data, non-parametric statistics were used. Spearman correlation coefficients (r) were calculated to analyse the extent to which the five IPA "life impact areas" correlated to upper extremity functioning (ARAT and kinematics) and activity performance (independence) in ADL. The strength of the correlation was interpreted as 0.00–0.25 (very low), 0.26–0.49 (low), 0.50–0.69 (moderate), 0.70–0.89 (high), and 0.90–1.00 (very high)^36^.

### Ethics approval and consent to participate

The study was approved by the Swedish Ethical Review Authority (registration number 408-17). We certify that all applicable institutional and governmental regulations concerning the ethical use of human volunteers were followed during the course of this research.

## Results

Of the 411 medical records reviewed, 216 individuals with SCI were considered as potential participants for the current study. Of those who responded to the call (n = 134), 32 were interested in participating, and 25 met the inclusion criteria and were enrolled. The mean age of the study population was 58.4 years (range, 26–81), 72% were men, the mean time since injury was 17.5 years (range, 1–53), 68% received assistance, and the majority had received home and car adaptations. The background and clinical characteristics of the participants are presented in Table 1.

**Table 1:** Characteristics of participants with SCI (n = 25).

Characteristics	Median or n	Q1; Q3 or %
Age, years	55	49.5; 71
Years since injury	9	5.5; 33
Male	18	72%
BMI	24	22; 28
Paid or voluntary work	16	64%
Single family home	8	33%
Receiving assistance	17	68%
Personal assistance	6	24%
Home care	2	8%
Assistance from a family member	9	36%
No assistance	8	32%
Adapted home	19	76%
Adapted car	14	56%
Level of spinal cord injury		
Cervical	17	68%
Thoracic	8	32%
Completeness of SCI		
AIS type A, B	14	56%
AIS type C, D	11	44%
Severity of SCI		
C1–C4 AIS type A, B, C	5	20%
C5–C8 AIS type A, B, C	5	20%
T1–T12 AIS type A, B, C	7	28%
AIS type D	8	32%
Hand surgery	8	32%
Upper extremity activity capacity, ARAT (0–57)	52	37.5; 57
Kinematics		
Movement time, s	7.2	5.7; 8.9
Smoothness, number of movement units	8.0	6.1; 10.6
Wrist angle, degrees	31.1	25.8; 43.6
Trunk displacement, cm	5.9	1.7; 8.2
SCIM-self-care (0–20)	18	10.5; 18.5
SCIM-respiration & sphincter management (0–40)	27	20; 34
SCIM-mobility (0–40)	18	11; 29.5
SCIM-total (0–100)	65	49.5; 80.5

AIS type A (complete injury), B (sensory incomplete, but motor complete), C (incomplete, muscle grade 0-2), D (incomplete, muscle grade ≥3): American Spinal Injury Association (ASIA) Impairment scale; ADL, Activities of Daily Living; ARAT, Action Research Arm Test; BMI, Body Mass Index; SCIM, Spinal Cord Independence Measure; Q1 and Q3, 1st and 3rd quartiles.

### Perceived autonomy in life areas

**Figure 1:**
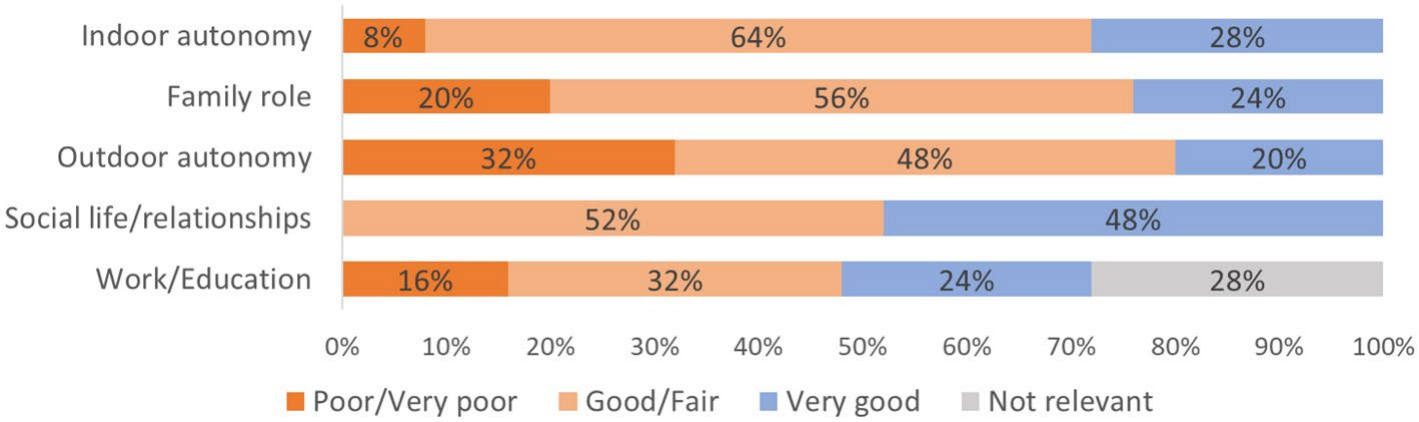
The autonomy and participation in five “life impact areas” as reported by the participants with SCI.

Participants reported autonomy restrictions in all “life impact areas” (Fig. 1). Most participants reported restrictions in in outdoor autonomy (80%), family role (76%) and indoor autonomy (72%). About half of the participants perceived restrictions in social life and relationships (52%) as well as work and/or education (48%) areas. The outdoor autonomy was reported to be poor or very poor by 32%, followed by family role (20%), work and education (16%) and indoor autonomy (8%) domains. None of the participants found the autonomy in social life and relationships poor or very poor and about half reported no restrictions in this domain. Within the social life and relationships domain, however, one question was more commonly reported to be poor or very poor compared to other questions. The “chances of having an intimate relationship” was reported to be poor or very poor by 56% of participants, fair or good by 36%, and only 4% selected the option very good. One in five participants (20%) perceived that their chances to living life the way they wanted were poor or very poor (question 10) and about the same proportion perceived the autonomy, in general, to be very good (16%).

**Figure 2:**
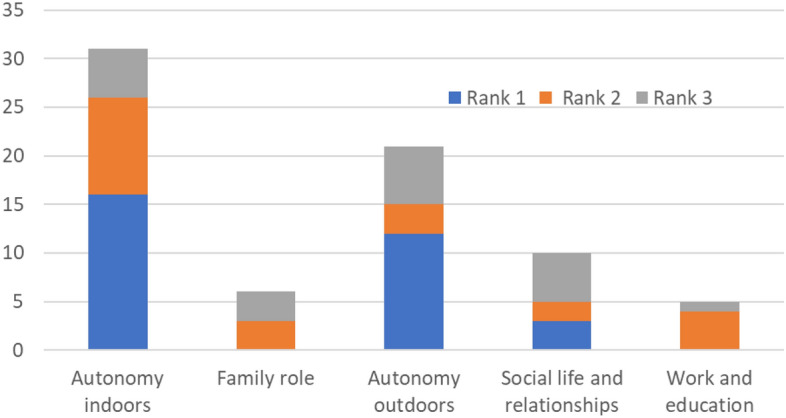
Participants’ rankings of the three top problems due to the spinal cord injury categorised in the “life impast areas” of autonomy and participation.

Among the three main problems as reported by the participants, the highest ranked were linked to the indoor and outdoor autonomy, followed by social life, family role and work/education autonomy areas (Fig. 2). Additional problems impacting autonomy, as reported by the participants, included aspects of acceptance, human values, mental health, physical symptoms, sexual functions, medical issues, environmental factors, and factors related to the healthcare system.

### Correlations between autonomy, upper extremity functioning and independence in ADL

Movement time during a purposeful daily task execution (r = 0.63), independence in self-care (r = 0.72), mobility (r = 0.59) and overall ADL (r = 0.59) were all moderately correlated to indoor autonomy (Table 2). Statistically significant but lower level correlations were found between movement smoothness and indoor autonomy (r = 0.49); between social life autonomy and self-care (r = 0.50), mobility (r = 0.44), overall ADL independence (r = 0.40) and upper extremity capacity (r = 0.41); and between outdoor autonomy and self-care (r = 0.48, Table 2 and 3). In reference to ICF, the autonomy and participation showed strongest correlation with independence in self-care activities (r = 0.72, activity performance), followed by the time to perform the drinking task (r = 0.63, upper extremity function).

**Table 2:** Spearman correlation coefficients between autonomy and participation life impact areas and upper extremity functioning.

Autonomy impact areas	UE activity capacity	Kinematics			
		Movement time	Smoothness	Wrist angle	Trunk displacement
Indoors	− 0.33	0.63**	0.49*	0.06	0.24
Family role	− 0.04	0.12	0.04	− 0.11	− 0.15
Outdoors	− 0.14	0.14	0.18	− 0.18	− 0.20
Social life and relationships	− 0.41*	0.25	0.35	0.23	− 0.15
Work/education (n = 18)^a^	− 0.30	0.26	0.35	0.16	− 0.04

*UE* upper extremity.

^a^7 participants were retired.

*p < 0.05; **p < 0.01 Correlations ≥ 0.50 are marked with bold;

**Table 3:** Spearman correlation coefficients between autonomy and particpation life impact areas and activity performance (independence) in activities of daily living.

Autonomy impact areas	Independence according to SCIM			
	Self-care	Respiration/sphincter management	Mobility	Overall ADL
Indoor autonomy	− 0.72**	− 0.28	− 0.59**	− 0.59**
Family role	− 0.33	0.06	− 0.25	− 0.20
Outdoor autonomy	− 0.48*	0.09	− 0.34	− 0.26
Social life and relationships	− 0.50*	− 0.08	− 0.44*	− 0.40*
Work/education (n = 18)^a^	− 0.24	0.43	− 0.29	− 0.07

*SCIM* spinal cord independence measure, *ADL* activities of daily living.

*p < 0.05; **p < 0.01 Correlations ≥ 0.50 are marked with bold.

^a^7 participants were retired.

## Discussion

The findings of this study showed that the majority of participants experienced restrictions in autonomy and participation across multiple life areas after the SCI. About one in five participants perceived the autonomy in general to be poor or very poor. The indoors and outdoors autonomy, reflecting the restrictions experienced in self-care and mobility in and outside of the house, were the most affected life areas. The family role, reflecting a limited chance to contribute to various household activities and tasks as a family or group member, was also one of the most impacted areas after the SCI. Autonomy in social and work life was affected in about half of the participants. The top three injury-related problems as reported by the participants were also most commonly linked to the indoor and outdoor autonomy life areas. The results of this study also showed that the autonomy in indoor activities and social life were to large degree correlated to upper extremity functioning and independence in self-care and mobility. These associations indicate a clear linkage between these domains and underlines the role of upper extremity functioning in common daily activities as well as in participation and autonomy in various social contexts.

In reference to the ICF, the relationships between the autonomy in major life areas and the activity performance in ADL were expected to be stronger compared to upper extremity function as measured by the kinematics. This hypothesis was true, but even kinematic measures of movement time (r = 0.63) and movement smoothness (r = 0.49) showed statistically significant moderate correlations. The analysis revealed that the perceived restrictions in indoor autonomy were correlated to slower movement times and poorer movement smoothness during the drinking task performance. These findings are unique and to our knowledge the relationships between the autonomy and upper extremity movement performance as measured by kinematics has not been reported previously. These findings provide some new insights on the importance of upper extremity functioning in participation and autonomy in life. The poorer upper extremity functioning, and particularly the prolonged movement times and poorer movement quality can influence the degree of independence in self-care and mobility (which to large degree is dependent on upper extremity functioning), which in turn can influence participation and autonomy particularly in common indoor and social life activities. Previous research in people with SCI, showing significant correlations between the independence in self-care and upper extremity functioning measured by kinematics or clinical scales, supports this potential linkage^11,12,14^. In a previous study, kinematic measures of movement time, movement smoothness and wrist angle explained 82% of the variance in ARAT in people with SCI, which further supports the potential linkage to the upper extremity function. In the current study, significant associations to autonomy and participation were found for movement time and movement smoothness, but not for wrist angle or trunk displacement. This finding proposes that specific joint configurations and movement strategies used in activity performance are less determining in terms of participation and autonomy. On the other hand, the slower movement times and poorer movement quality (movement time and smoothness) seem to influence the autonomy and participation particularly in self-care and indoor mobility.

The top three problems due to the SCI, as ranked by the participants, were most commonly linked to the indoor and outdoor autonomy, which lines well with the participants' responses to the questions regarding the major life impact areas. This finding emphasises the importance of having satisfactory functioning in- and outside of the house including self-care to reach satisfactory autonomy. These life aspects are likely connected to person’s living arrangements, assistance received and contextual environmental factors. In our cohort, despite that majority were living in adapted home (76%), received assistance (68%) or drove an adapted car (56%), the in- and outdoor autonomy was still considered problematic by many. The autonomy in family role life area showed to be restricted in 76% of participants, still, none of the participants ranked it as the number one problem. Likewise, the autonomy in work and education was never ranked first, although five participants had listed it as the second or third problem. This result may reflect the tendency to prioritize the basic needs, such as self-care and mobility, over the family role, work and education.

None of the participants perceived the autonomy in social life and relationships as poor or very poor when the median score for this domain was calculated (Fig. 1). The responses to individual questions revealed, however, that for the question on “my chances of having an intimate relationship”, 56% of participants selected the options poor or very poor. So even when the autonomy in social life and relationships was perceived as fair or good by most when considering the different aspects of this domain, the autonomy in this specific life aspect was perceived as poor or very poor by many. This highlights the need to specifically address the autonomy in intimate relationships even when the general autonomy in social relationships might be perceived as good.

The results from the current study can have important implications to the SCI rehabilitation. Firstly, the majority of individuals with cervical or thoracic SCI can perceive restrictions in autonomy and participation^37^, which suggests that these aspects should be addressed in SCI rehabilitation. Secondly, the independence in ADL and mobility, which are often in focus after SCI, seem also to be important for self-perceived autonomy and participation. Thirdly, the understanding of the linkage between the upper extremity functioning and autonomy/participation in indoor activities in various social contexts, can be used to guide the joint goal setting and planning of interventions in rehabilitation together with people with SCI.

### Strengths and limitations

This study covered a wide range of life areas relevant to the participation and autonomy in people with SCI. The results of the current study are applicable to people with cervical or thoracic SCI living in Sweden. In our cohort, the majority of the participants lived in adapted housing, received assistance and drove an adapted car. The role of the contextual factors influencing autonomy were not specifically investigated in the current study. Future studies in countries with different healthcare systems could provide further insights into perceived autonomy in different geographical or social environments. In addition, the influence of upper extremity functioning on autonomy should be further investigated in other cohorts. The literature reporting autonomy in people with SCI is very scare, and therefore not many comparisons with other studies could be made. The sores of the IPA questionnaire are also often reported as mean values^37^, which masks some of the finer nuances of the responses. In general, the results in our cohort are in line with previous studies that have reported mean values of the five autonomy domains to be between fair (2) and poor (1) categories in people with SCI^37^. In our data (Fig. 1), the score 0 (no restrictions in autonomy) were reported by 29%, the scores 1 and 2 (good and fair) by 50%, the scores 3 and 4 (poor and very poor autonomy) by 15% of the participants, in average. Other limitations of the study were the relatively small sample size and that several potential other factors potentially influencing the autonomy and participation e.g. cognitive functions^38,39^, environmental factors^40,41^, were not collected.

## Conclusions

The current study provided new insights into understanding of perceived autonomy in major life areas after SCI and its relationships to the upper extremity functioning and independence in ADL. Autonomy restrictions were reported by the majority of participants in all major life areas. Indoor and outdoor autonomy as well as the family role showed the largest extent of restrictions. The domains of indoor and social life autonomy were also influenced by the independence in self-care, mobility and upper extremity functioning. The findings of this study emphasize the importance of including the aspects of autonomy and participation to individual goal setting and treatment planning after SCI. The role of upper extremity in different life impact areas of autonomy and participation should also be considered in clinical decision-making.

### Supplementary Information

Supplementary Information.

## Data Availability

The data that support the findings of this study are available via corresponding author on a reasonable request.

## Abbreviations

ADL: Activities of Daily Living
ASIA: American Spinal Injury Association
AIS: ASIA Impairment scale
ARAT: Action Research Arm Test
ICF: International Classification of Functioning, Disability and Health
IPA-E: Impact on Participation and Autonomy, the English version
ISCoS: International Spinal Cord Society
ISNCSCI: International Standards for Neurological Classification of Spinal Cord Injury
SCI: Spinal Cord Injury
SCIM-III: Spinal Cord Independence Measure version III
STROBE: Strengthening the Reporting of Observational studies in Epidemiology
