# Supplementary material for: Participation and autonomy, independence in activities of daily living and upper extremity functioning in individuals with spinal cord injury
Source: Sci Rep. 2024 Apr 20;14:9120. doi: 10.1038/s41598-024-59862-2 (PMC11032406; doi:10.1038/s41598-024-59862-2)
Supplement: Supplementary file 1 — Supplementary Information. [file 41598_2024_59862_MOESM1_ESM.docx]

**Supplementary table:** The items in the Impact on Participation and Autonomy (IPA) English version categorised according to “life impact areas” and the domains of the IPA.

| **Life impact areas** | **Stated questions:**  **“My chances of….. I want to are...”** | **The 9 domains of the IPA** |
| --- | --- | --- |
| **Indoor autonomy** | getting around in my house where, when, and the way… | Mobility |
|  | getting up and going to bed when | Mobility |
|  | getting washed and dressed the way | Self-care |
|  | going to the toilet when | Self-care |
|  | eating and drinking when | Self-care |
| **Family Role** | contributing to looking after my home the way | Household activities |
|  | getting light/heavy tasks done around the house or in my house and garden, either by myself or by others, the way | Household activities |
|  | getting housework done, either by myself or by others, when | Household activities |
|  | fulfilling my role at home as | Household activities |
|  | choosing how I spend my own money | Finance |
| **Outdoor autonomy** | visiting relatives and friends when | Mobility |
|  | going on the sort of trips and holidays | Mobility |
|  | using leisure time the way | Leisure |
|  | seeing people as often as | Social life |
| **Social Life and relationships** | talking to people close to me on equal terms | Social life |
|  | The quality of my close relationships | Social life |
|  | The respect I receive from people close to me is | Social life |
|  | My relationships with acquaintances are | Social life |
|  | The respect I receive from acquaintances is | Social life |
|  | having an intimate relationship are | Social life |
|  | helping or supporting people in any way are | Helping others |
| **Work and**  **Education** | getting/keeping a paid or voluntary job that | Paid or voluntary work |
|  | doing my paid or voluntary work the way | Paid or voluntary work |
|  | My contacts with other people at my paid or voluntary work are | Paid or voluntary work |
|  | …achieving or keeping the position that I want, in my paid or voluntary work, | Paid or voluntary work |
|  | …getting different paid or voluntary work | Paid or voluntary work |
|  | …getting the education or training | Education and Training |
